# Ideal parameters for femto-second laser-assisted anterior capsulotomy: Animal studies

**DOI:** 10.1371/journal.pone.0190858

**Published:** 2018-01-05

**Authors:** Min-Ji Kang, Yong-Eun Lee, Jun-Sub Choi, Choun-Ki Joo

**Affiliations:** 1 Department of Ophthalmology and Catholic Institute for Visual Science, College of Medicine, The Catholic University of Korea, Seoul, Republic of Korea; 2 Seoul St. Mary’s Hospital, Seoul, Republic of Korea; National Eye Institute, UNITED STATES

## Abstract

In femtosecond laser-assisted cataract surgery, the parameter such as horizontal spot spacing and energy level can be adjusted. Although there have been several studies reported on various laser systems, showing the effects of varying energy levels and horizontal spot spacing on lens capsulotomy cut edges, none have been reported on the Catalys laser system (Abbott Medical Optics, Inc., Santa Ana, CA). The aim of this study is to evaluate, using scanning electron microscopy (SEM), the quality of the cut edges of the laser lens capsulotomy obtained using the Catalys Laser System, using different horizontal spot spacing and energy levels, and to determine the ideal parameters based on SEM results. Fifty rabbit capsulorhexis specimens from a femtosecond laser with different spot spacing and energy settings were divided into five groups randomly. Spot spacing was 3 um and laser pulse energy was 4 uJ in group 1. The respective values were 5 um and 2 uJ in group 2, 5 um and 4 uJ in group 3, 5 um and 6 uJ in group 4, and 7 um and 4 uJ in group 5. All samples were evaluated using SEM to compare the number of tags per capsulotomy and the laser emission time. Group 1 had a significantly lower tag formation than groups 3 and 5 (*P* = 0.042 and 0.021, respectively). Although the laser emission time increased about 1.5 sec as the spot spacing increased from 3 to 7 um, the quality of the cut was smoother in group 1 because of overlapping effect of photodisruption cavities. There was no significant difference between groups 2, 3 and 4 at different laser energy settings. In an ex-vivo study, samples from an energy setting of 10 uJ showed increased irregularity and damage. The degree of irregularity was higher at increasing spot spacing and laser energy settings, with abundant tag formation. Dense spot spacing with low-energy settings provide a better cut quality, which is probably correlated with the reduction in anterior capsular tear complications.

## Introduction

Femtosecond (FS) laser is a pulsed laser device that emits a laser beam with a short pulse duration in the domain of femtoseconds. It operates as a cutting knife by essentially vaporizing tissue. The tightly focused laser energy creates plasma and then a cavitation bubble, separating the tissue [1]. Due to the convenient user interface, a surgeon can change many parameters, such as horizontal spot spacing or laser energy, within their limitations during preoperative planning.

FS laser anterior capsulotomy produces a perfectly round capsulotomy in a few seconds. Overall, FS laser capsulotomies have been shown to be better centered than manual continuous curvilinear capsulorhexis (CCC), with highly predictable sizes [2–4].

The surgical goal is the easy removal a free floating capsulotomy specimen without complications, such as anterior capsular tear. Rough capsule edges and tags have been described [5, 6], suggesting that microstructural integrity might be linked to an increase in anterior capsule tears [7]. A greater incidence of capsular tears using the FS laser platform compared to classic phacoemulsification surgery, even after the initial learning curve expected with the technology has been reported [8].

Overall, variations in laser settings affect the morphology of capsulotomy cut edges [6, 7, 9, 10]. In this study, we compared number of microscopic tags from the capsulotomy edge specimens and duration of laser emission for assisted capsulotomy performed using the Catalys Precision Laser System (Abbott Medical Optics, Inc., Santa Ana, CA) by scanning electron microscopy (SEM). We describe the ideal parameters for horizontal spot spacing and laser energy to achieve better microstructural regularity of capsulotomy after FS laser-assisted cataract surgery (FLACS).

## Subjects and methods

### *In vivo* animal experiments

The study was conducted in accordance with the ARVO Statement for the Use of Animals in Ophthalmic and Vision Research, and approved by the Institutional Animal Care and Use Committee (IACUC) at the College of Medicine, The Catholic University of Korea. Sixty New Zealand white rabbits of either gender aged 10 to 12 weeks weighing 2.5 and 3.0 kg were used (Orient Bio Inc., Sungnam, South Korea). Rabbits were deeply anesthetized by intramuscular injection of tiletamine (5 mg/kg body weight) and zolazepam (5 mg/kg body weight). Also, proparacaine (Alcaine; Alcon, Fort Worth, TX) was used for topical anesthesia to prevent and minimize possible movements. Excessive sodium pentobarbital anesthesia was used for rabbit sacrifice. All of the efforts were made to minimize suffering. Anterior capsule specimens from FS laser-assisted capsulotomy were obtained from rabbits. Rabbits in the laser group, they were created using the Catalys system. Anterior capsules from the control group included specimens obtained following manual CCC. All surgeries were performed by a single surgeon (CKJ).

### Settings of parameter

The laser parameters included the capsule diameter, laser pulse energy, spot spacing and layer separation parameters. These settings were optimized by the manufacturer as 5 um and 4 uJ, respectively, but within the limited range, spot spacing size was changed as 3, 5, and 7 um, and the laser pulse energy setting was set at 2, 4, and 6 uJ. A total of 60 eyes (60 rabbits) were studied from May 2014 to December 2014. Capsulotomy specimens were removed using rhexis forceps from 50 eyes. Ten consecutive phacoemulsification CCC capsule was collected as a control for SEM.

### Scanning electron microscopy

Specimens were placed in a sterile container filled with fixative and prepared for SEM immediately after surgeries. Samples were fixed in 2.5% glutaraldehyde in sodium cacodylate buffer, dehydrated using a series of ethanol solutions of increasing concentration [3], and critical-point dried according to standard SEM protocol [11]. After drying, samples were coated with gold and mounted on metal plates with double-sided carbon tape. Images of capsular edges were obtained at various magnifications.

### Groups

Rabbits were randomized to group 1 (spot spacing 3 um, laser pulse energy 4 uJ), group 2 (5 um and 2 uJ, respectively), group 3 (5um and 4 uJ, respectively), group 4 (5 um and 6 uJ, respectively), and group 5 (7 um and 4 uJ, respectively). In one eye, manual CCC and conventional phacoemulsification was performed. Capsulotomy diameter was fixed at 5.2 mm, the anterior/posterior safety zone for laser treatment was ± 300 um, and layer separation was set to 10 um.

### *Ex vivo* animal experiments

We used ex-vivo rabbit eyes and our own prototype FLACS apparatus capable of an extraordinary energy setting to explore the effect of very high energy on adjacent tissues. Our prototype was necessary because pulse energy over 10 uJ is not provided in the Catalys system for safety. The energy was applied as 4uJ, 7uJ and 10uJ in 3 eyes each and spot spacing was set as 5um.

Analyses of the laser emission time for capsulotomy and cross section of capsulotomy samples using SEM were performed. As prior studies reported, tags can be found using magnified examination. So we counted the number of tags per capsulotomy in certain conditions and compared the number of tags and laser emission time between the groups. Cause of smoother cut surface from calculated spot size and cause of anterior capsular tear in relation with tag were also explored.

### Statistical analysis

Comparisons between the five groups were performed using the Mann-Whitney U test. SPSS 18.0 software (SPSS, Chicago, IL) was used for all analyses, with P values < 0.05 indicating significance.

## Results

### *In vivo* animal experiments

#### Horizontal spot spacing

**Fig 1:**
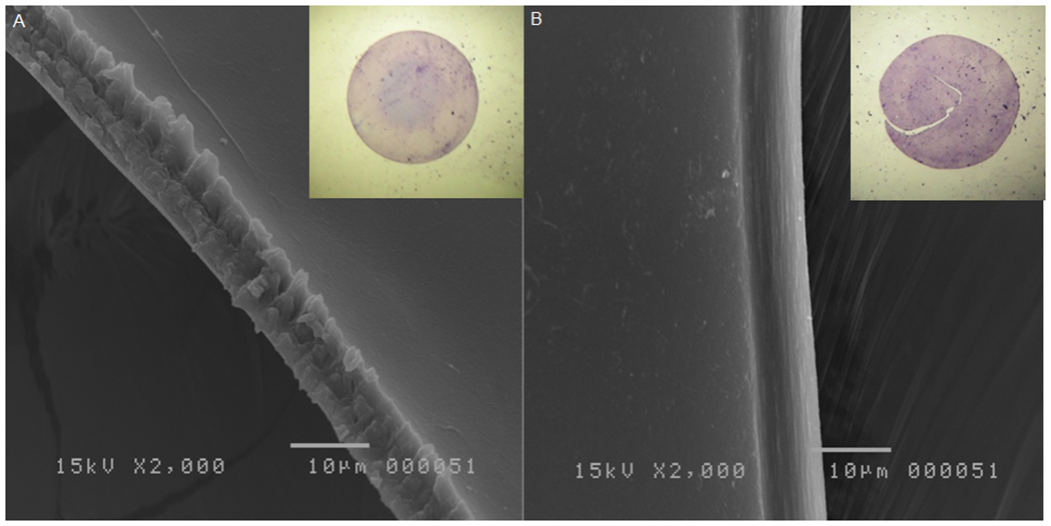
Capsulotomy-cut feature in high resolution SEM. A. FLACS; High resolution SEM shows serrated, resembling a micro can-opener structure (original magnification, x2000). B. Manual CCC; Smooth surface of cut edge (original magnification, x2000).

Postage-stamp perforations were present in every FLACS specimen (Fig 1A), but the manual capsulorhexis specimens did not show any imperfections (Fig 1B).

**Fig 2:**
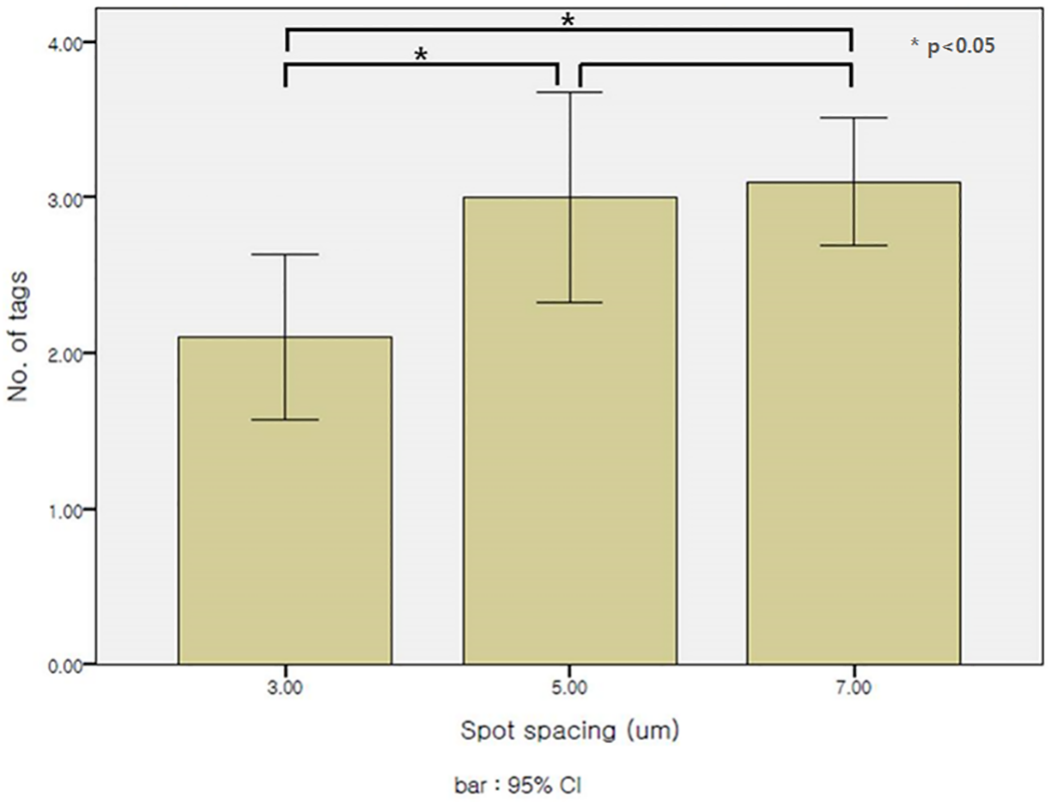
Number of tags formation per capsulotomy at various horizontal spot spacing setting. Number of tags according to spot spacing was 2.10 ± 0.74 at 3 um, 3.00 ± 0.94 at 5 um, and 3.10 ± 0.57 at 7 um. Tag formation was statistically significantly less In 3 um setting.

**Fig 3:**
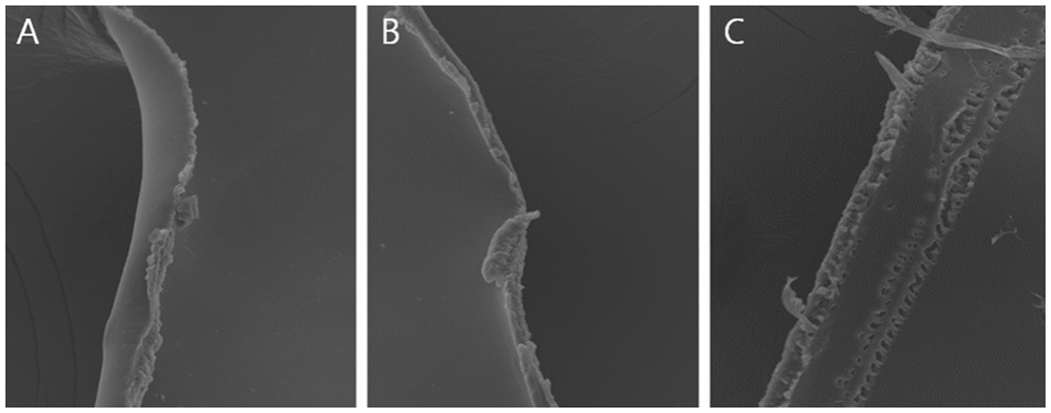
Tag formation according to spot spacing. When spot spacing is set at 3um (A) and 5um (B), tag is small and not prominent, but if the spot spacing is enlarged to 7um (C), irregular cut edge with multiple tags are prominent (original magnification, x500).

Number of tags according to spot spacing was 2.10 ± 0.74 at 3 um, 3.00 ± 0.94 at 5 um, and 3.10 ± 0.57 at 7 um. In group 1 at the 3 um setting, tag formation was statistically significantly less than for groups 3 and 5 (P = .042) (Fig 2). But laser emission time was increased from 1.12 ± 0.04 sec to 2.57 ± 0.05 sec as the spot spacing was decreased to 3 um. It was also statistically different (P < .001) (Table 1). Time difference between 3 um and 7 um settings was less than 1.5 sec, which was not clinically important. So, the ideal spot spacing with lesser tag and smoother edge, with a slight time lag, was 3 um (Fig 3).

**Table 1:** Tag formation and laser emission time according to spot spacing.

Spot spacing (um)	No. of tags per capsulotomy	P value	Laser emission time for CCC (sec)
3	2.10 ± 0.74		2.57 ± 0.05
5	3.00 ± 0.94	0.042	1.59 ± 0.03
7	3.10 ± 0.57	>.999	1.12± 0.04

laser emission time was increased from 1.12 ± 0.04 sec to 2.57 ± 0.05 sec as the spot spacing was decreased to 3 um.

**Fig 4:**
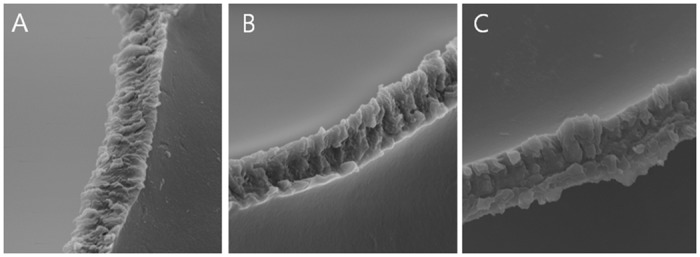
Cutting edges in femtosecond laser-assisted capsulotomy with a smoother margin using 3 um (A) compared with 5 um (B) and 7 um (C) (original magnification, x2000). The surface at 3 um was smoother (Fig 4A) compared to 5 um (Fig 4B) and 7 um (Fig 4C).

The spot spacing at 3 um was preferred over the manufacturer’s recommendation of 5 um. The surface at 3 um was smoother (Fig 4A) compared to 5 um (Fig 4B) and 7 um (Fig 4C). The reason for the smoother surface at 3 um was the size of the spots formed from the laser optic system. In this laser flatform, each photodisruption spot diameter was estimated to be 2.69 ± 0.41 um; if a surgeon set the spot spacing at 3 um, nearby spots overlapped and a smoother cut edge could be achieved. A spot spacing exceeding 3 um prevented overlap, resulting in a can-opener-like surface.

#### Laser energy level

Number of tags per capsulotomy was 3.00 ± 1.05 at 2 uJ, 3.00 ± 0.94 at 4 uJ, and 2.97 ± 0.93um at 6 uJ. The number of tag formation and laser emission time according to laser energy was not significantly different between the groups (*P* > .999, Table 2).

**Table 2:** Tag formation and laser emission time according to various laser energy level.

Laser energy (uJ)	No. of tags per capsulotomy	*P* value	Laser emission time for CCC (sec)
2	3.00 ± 1.05		1.58 ± 0.04
4	3.00 ± 0.94	>.999	1.59 ± 0.03
6	2.97 ± 0.93	>.999	1.60 ± 0.08

The number of tag formation according to laser energy was not significantly different between the groups.

### *Ex vivo* animal experiments

**Fig 5:**
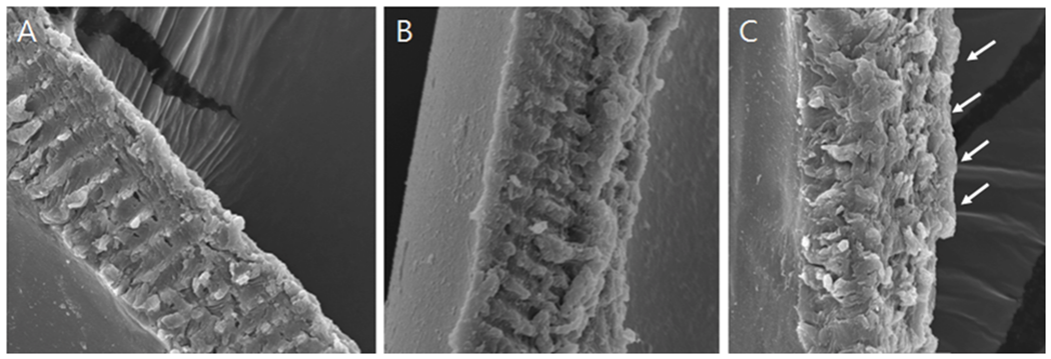
Excessive laser energy results in adjacent tissue damage. Laser energy setting at 4 uJ (A) and 7 uJ (B), cutting edge was relatively well-preserved. Due to high energy, partly destroyed edge was prominent at 10 uJ (C, arrow) (original magnification, x2000).

We hypothesized that very high laser pulse energy would be harmful to adjacent tissues. Ex-vivo testing performed using our prototype FLACS equipment (which is not approved for human use) revealed increasingly damaged morphology with increasing laser energy level (Fig 5). At a laser energy under 7 uJ (Fig 5B), the cutting edge was relatively well-preserved. A partly destroyed edge was prominent at 10 uJ indicating that very high energy can damage adjacent tissues and cause poor prognosis postoperatively.

## Discussion

At present, the expectation of an ideal refractive outcome after cataract surgery is increasing, especially after implantation of premium intraocular lenses, such as multifocal, toric and accomodative models. The FS laser has been used for cataract surgery with the goal of improving the efficacy and safety of the cataract procedure [12]. The FS laser optimizes capsulotomy, creating a round, continuous, sharp-edged capsulorhexis that results in increased tensile strength [2–4]. Another study reported that laser capsulotomy is compromised by postage-stamp perforations and additional aberrant pulses, possibly because of fixational eye movements [7].

This study evaluated the ideal spot spacing and laser energy to minimize tag formation and make smooth cut edge to avoid unwanted complications, such as radial tear and adjacent tissue damage. Although many studies have compared FS laser-assisted capsulotomy and manual CCC, this is the first study to address the ideal parameter settings for the Catalys laser system. We compared the time for anterior capsulotomy at different settings. Tags were significantly less at the 3 um spot spacing setting than at 5 or 7um. Although the laser emission time for capsulotomy increased as the spot spacing decreased, the total time difference was only 1.5 seconds, which is not clinically important. So we recommend dense spot spacing during CCC in FLACS to achieve ideal edge shape. Benefits include lower laser energy resulting in less damage to the cut edge. Higher energy, such as 10 uJ, could be harmful.

Because published evidence for ideal parameters for FLACS is limited, especially with the Catalys laser system, we wanted to determine evidence-based parameter guidelines for users.

There were two cases of anterior capsular tear (2/50). SEM analyses proved that the microscopic tags were the cause of complications. It was a slightly higher rate than prior studies and surveys of Catalys users [7, 13], but we have to consider the specific conditions of our mature cataract patient (one case) and influence of a learning curve effect, as previously identified [2].

In the current study, we used SEM to find out the morphology and quality of the capsulorhexis cut obtained with the FS laser at different spot spacing and energy settings during cataract surgeries. High-magnification images revealed postage-stamp like irregularities with small tags. Almost all of the capsulotomy specimens were free-floating in the operative field. Nonetheless, a surgeon should keep the microstructural integrities in mind to prevent anterior capsular radial tear.

In conclusion, SEM evaluation of the capsulorhexis from FLACS found better surface-cut quality under denser spot spacing with mild adjacent tissue damage at the lower energies settings. To exploit the advantages of FLACS, a surgeon remain cognizant of microscopic tags after removal of the capsulotomy disk to prevent complications, such as anterior capsular tear.

## Supporting information

S1 Checklist Checklist of ARRIVE guidelines. (PDF)

S1 Table Tag formation and laser emission time according to spot spacing. (XLSX)

S2 Table Tag formation and laser emission time according to various laser energy level. (XLSX)
